# Loss of RANBP3L leads to transformation of renal epithelial cells towards a renal clear cell carcinoma like phenotype

**DOI:** 10.1186/s13046-021-01982-y

**Published:** 2021-07-07

**Authors:** Dmitry Chernyakov, Alexander Groß, Annika Fischer, Nicola Bornkessel, Christoph Schultheiss, Dennis Gerloff, Bayram Edemir

**Affiliations:** 1 Department of Medicine, Hematology and Oncology, Martin Luther University Halle-Wittenberg, Halle (Saale), Germany; 2 Department of Dermatology and Venereology, Martin Luther University Halle-Wittenberg, Halle (Saale), Germany; 3 Current address: Klinik für Innere Medizin IV, Hämatologie und Onkologie, Universitätsklinikum Halle (Saale), Halle (Saale), Germany

**Keywords:** Renal cell carcinoma, Tumor progression, Tumor suppressor, CRISPR/Cas9, Hyperosmolality

## Abstract

**Background:**

Renal cell carcinomas (RCC) are characterized by the deregulation of several hundred hyperosmolality-responsive genes. High expression of a subset of these genes including the Ran binding protein 3 like (*RANBP3L*) is linked to a favorable prognostic outcome in RCC. However, the cellular function of RANBP3L remains largely unknown.

**Methods:**

We used CRISPR/Cas9-mediated gene editing to generate functional deletions of the *Ranbp3l* and nuclear factor of activated T cells 5 (*Nfat5*) gene loci in a murine renal cell line. The NFAT5-KO cells were used to assess the regulation of *Ranbp3l* by NFAT5 using immunofluorescence, RNA-Seq and promoter assays. RANBP3L-deficient cells were analyzed for changes in cell morphology, proliferation, migration and colony-forming capacity using immunofluorescence and live cell imaging. RANPB3L-dependent changes in gene expression were identified by RNA-Seq.

**Results:**

We show that NFAT5 directly regulates *Ranpb3l* under hyperosmotic conditions by binding its promoter. Functional analysis of RANBP3L-deficient cells revealed a loss of epithelial structure, an increased cell migration behavior and colony forming capacity, accompanied by massive alterations in gene expression, all of which are hallmarks for tumor cells. Strikingly, a RANBP3L dependent signature of 60 genes separated samples with clear cell carcinoma (KIRC) from papillary (KIRP), chromophobe renal carcinoma (KICH) and healthy tissue.

**Conclusions:**

Loss of RANBP3L induces a tumor like phenotype resembles RCC, especially KIRC, on the morphological and gene expression level and might promote tumor development and progression. Therapeutic reconstitution or elevation of osmoregulated *RANBP3L* expression might represent a novel treatment strategy for RCC or KIRC.

**Supplementary Information:**

The online version contains supplementary material available at 10.1186/s13046-021-01982-y.

## Background

Renal cell carcinoma (RCC) refers to a heterogeneous group of cancers originating from the renal epithelium which comprise the major subtypes clear cell RCC (KIRC, accounting for 70–80% of cases), papillary RCC (KIRP, 10–15% of cases), and chromophobe RCC (KICH, 3–5% of cases) [1]. One major risk factor for developing RCC includes loss or inactivation of the von Hippel–Lindau (*VHL*) tumor suppressor gene [2]. To date, several VHL mouse models have been generated to recapitulate important aspects of RCC pathobiogenesis and established VHL as key regulator of hypoxia inducible factors (HIFs) as well as central signaling networks like PI3K/AKT, Notch, or NFκB-signaling pathways [3–5]. The renal deletion of VHL is associated with increased medullary vascularization (IMV) leading to the excretion of highly diluted urine most likely because IMV alters the salt uptake from the interstitium which results in the disruption of the hyperosmotic gradient necessary for urinary concentration [5]. The key transcription factor for inducing the primary cellular (hyper) osmoregulatory program is the nuclear factor of activated T-cells 5 (NFAT5; also known as tonicity enhancer binding protein, TonEBP) [6, 7]. NFAT5, a pleiotropic stress protein also mediates metabolic reprogramming in several immunological disorders and is associated with cancer development and progression in numerous entities [8–13]. A recent example for this association is the microRNA-mediated decrease of *NFAT5* transcript levels in Caki-2 cells resulting in the downregulation of osmoprotective NFAT5 target genes, a pattern also observed in clinical KIRC specimen [8].

In line with this data, we have recently shown that the hyperosmotic environment is an important regulator of several kidney-specific genes in primary cultured renal inner medullary collecting duct cells (IMCD) [14]. The expression of these osmolality-affected genes is inversely regulated in KIRC samples. Genes induced by hyperosmolality showed a reduced expression and genes suppressed by hyperosmolality an induced expression which correlates with patient overall survival [15]. We have also shown that the loss of VHL in murine cells interferes with the expression of this osmo-adaptive gene set and induces a gene expression pattern that is in summary unfavorable for patients with RCC [16]. One of these genes is the Ran binding protein 3-like (*Ranbp3l*).

RANBP3L belongs to a class of Ran-binding proteins characterized by the Ran-binding domain (RBD) [17]. The Ran (Ras-related nuclear protein) is a small GTPase of the Ras superfamily which plays a crucial role in nucleocytoplasmic transport and cell cycle progression [18]. The only published report functionally links RANBP3L to the TGF-β/BMP signaling pathway where it regulates the nuclear export of Smad proteins in mouse bone marrow-derived mesenchymal stem cells [19]. To date, a kidney-specific role of RANBP3L has not been reported and functional studies are limited.

Here we functionally characterize the role of RANBP3L in a renal mouse cell line model. We show that the expression of *Ranbp3l* is induced by NFAT5 under hyperosmotic conditions and that the loss of RANBP3L induces the transformation towards a renal cancer cell like phenotype, especially KIRC. This transformation is associated with a prognostic gene expression pattern that can be translated to human RCC samples. Based on these data, we propose that RANBP3L is a crucial regulator of epithelial integrity in renal cells and that its functional can promote cancer development and/or progression. For this reason, osmoregulated RANBP3L might represent a novel promising target for the development of treatment strategies for patients with KIRC.

## Material and methods

### Cell culture

HEK293T cells were obtained from the DSMZ-German Collection of Microorganisms and Cell Cultures and cultivated in Dulbecco’s Modified Eagle’s Medium (DMEM, 21885–108, Life Technologies, Carlsbad, California, USA) supplemented with 10% fetal bovine serum (FBS, P30–3031, PAN Biotech, Aidenbach, Germany) and 1% penicillin/streptavidin (P0781, Sigma Aldrich, St. Louis, Missouri, United States). The mpkCCD cell line was a kind gift of Prof. Mark Knepper [20]. These cells were cultivated in Dulbecco’s Modified Eagle’s Medium/Nutrient Mixture F-12 Ham medium (D8437, Sigma Aldrich, St. Louis, Missouri, USA) supplemented with 10% FBS and 1% penicillin/streptavidin. The Renal cell carcinoma cell lines 786–0, Caki-1 and Caki-2 were obtained from ATCC and cultivated in RPMI-1640 Medium (R8758, Sigma Aldrich) supplemented with 10% fetal bovine serum and 1% penicillin/streptavidin. Primary mouse IMCD cells were prepared from murine kidneys as described before [21, 22]. The cells were seeded in plates coated with collagen type IV (10376931, Thermo Fischer Scientific, Waltham, Massachusetts, United States) and cultivated in DMEM (FG 0435, Biochrom, Berlin, Germany) containing 1% penicillin and streptomycin, 1% nonessential amino acids (11140050, Thermo Fischer Scientific), and 1% Ultroser G (15950–017, CytoGen GmbH, Wetzlar, Germany). All cells were cultured at 37 °C and 5% CO_2_. The medium osmolality was adjusted to 600 mosmol/kg by the addition of 100 mM NaCl (71376, Sigma Aldrich) and 100 mM urea (U5378, Sigma Aldrich) to the corresponding medium.

### Oligos and primers

Primer for cloning, RT-qPCR primer and the PCR primer for amplification of the targeted locus were designed by NCBI Primer BLAST [23]. All Oligonucleotides were purchased from Biolegio B. V. (Nijmegen, Netherlands).

### CRISPR/Cas9-mediated knockout of NFAT5 and RANBP3L

Sequence design of the guide RNAs (gRNAs) targeting *Ranbp3l* and *Nfat5* was performed according to the CHOPCHOP online tool (https://chopchop.cbu.uib.no) [24]. Three different sequences were selected (Table S2 A, S3 A). The gRNAs targeting the murine *Ranbp3*l and *Nfat5* locus as well as a random non-targeting scrambled (Scr) gRNA were cloned into lentiCRISPRv2 (# 52961; Addgene, Watertown, MA, USA). The cloning was performed as described before [25]. Plasmid isolation was performed using GeneJET Miniprep Kit (K0502, Thermo Fisher Scentific). Isolated plasmids were tested for cloned gRNAs by Sanger sequencing (Eurofins Genomics, Ebersberg Germany) using human U6 primer (TabS. 4).

HEK293T were transfected with vectors from the ViraPower™ Lentiviral Packaging Mix (K4975–00, Thermo Fisher Scentific) and the CRISPR vector carrying one of the three gRNAs. Cells were incubated for 24 h. The cell media was replaced and the cells were incubated for additional 48 h for production of viral particles. The conditioned virus-containing medium was then removed, sterile filtrated and kept at − 20 °C.

The target mpkCCD cell line was seeded into 6-well cell culture dishes and cultivated to 40–50% confluency. Medium was removed and replaced by conditioned medium containing the viral particles and fresh medium in a 1:1 ratio. After 48 h, cells were trypsinized and cultured further on selection medium containing 2 μg/ml puromycin (P9620-10ML, Sigma Aldrich). Genomic DNA was isolated from transfected mpkCCD cells. Targeted regions were amplified by PCR using specific primers and the PCR products were purified using GenElute™ PCR Clean-up Kit (NA1020-1KT, Sigma Aldrich) and analyzed by Sanger sequencing (Eurofins Genomics GmbH, Eberberg, Germany).

Clonal isolations were performed by serial dilution in 96-well cell culture plates. Total DNA was isolated from the single clone and the targeted area was amplified by PCR and analyzed by Sanger sequencing. Sequencing data of single clones was then uploaded to the TIDE (Tracking of INDELs by Decomposition) (http://shinyapps.datacurators.nl/tide/) server and analyzed for specific frameshift mutations [26]. Single clones harboring frameshift mutations on both alleles were selected for validation by TOPO-TA (451,641, Thermo Fisher Scientific) cloning followed by Sanger sequencing with the M13 primer (TabS. 4) according to manufacturer’s instructions.

### RNA isolation, cDNA synthesis and RT-qPCR

RNA was isolated with the GenElute™ Total RNA prep Kit (RTN350-1KT, Sgima-Aldirch) according to manufacturer’s instructions. 1 μg of RNA was used for cDNA synthesis. The expression of target genes was determined by RT-qPCR using specific primers, separated by at least one intron on the corresponding DNA as described before [14]. Data acquisition was done with Bio-Rad CFX Manager 3.1 Software and quantified by 2^-∆∆CT^ method as described [27]. The sequences of the used primers are listed in supplemental Table 4.

### Western blot

Total protein was isolated from cells using Pierce® RIPA lysis and extraction buffer (89,900, Thermo Fisher Scientific) with protease inhibitor mix (40 μL/mL, 4,693,159,001, Sigma Aldrich). Protein lysates were separated by SDS-PAGE with 4–12% Novex™ Bis-Tris gradient gel (NP0322BOX, Thermo Fisher Scientific) using the Novex™ NuPAGE™ MES SDS running buffer (NP0002, Thermo Fisher Scientific). Separated proteins were then blotted onto 0,2 μm nitrocellulose membrane (10,600,001, GE Healthcare, Chicago, Illinois, USA). Unspecific binding sites were blocked with 5% BSA or milk solution. Primary and horse reddish peroxidase-coupled secondary antibodies were diluetd in milk or BSA solution according to manufacturer’s instruction and incubated for 1h at room temperature. Afterwards, the membrane was incubated with ECL™ Prime Western Blotting Detection Reagent (GERPN2236, GE Healthcare) and signals were detected on the ChemiDoc Imager detecting system (10,000,062,126, BioRad, Hercules, CA, USA).

### Immunofluorescence

Immunofluorescence was performed as described before [14]. Cells were seeded in 24-well plates on glass cover slips. Medium was removed and cells were fixed in 4% formalin. Unspecific binding sites were blocked by incubation with fishskin-gelatine (0.3% in PBS, G7765-1 L, Sigma Aldrich). First antibody was applied in gelatine solution and incubated at 37 °C for 1 h. Three wash steps (15 min) were performed with PBS and the cells were incubated for 1 h with the secondary Alexa-labeled antibody solution in PBS. The cell nucleus was stained with 4′,6-diamidino-2-phenylindole (DAPI, 268298, Merck Millipore, Burlington, Massachusetts, USA). The cells were washed three times with PBS (15 min) and mounted on glass slides with Fluoroshield histology mounting medium (F6182-20 ml, Sigma Aldrich). Images were taken on a Keyence BZ-8100E microscope (Keyence Corporation, Osaka, Japan) at 100, 200, 400x or 1000x magnification.

### Antibodies

We used following antibodies: anti-NFAT5 rabbit (ab3446, Abcam, Cambridge, UK), anti-GAPDH rabbit (14C10, Cell Signaling Technology, Danvers, Massachusetts, USA), Peroxidase AffiniPure F(ab’)_2_ Fragment Goat Anti-Rabbit IgG (111–036-047, Jackson Immunosearch, West Grove, Pennsylvania, USA), Goat anti-Rabbit IgG (H + L) Alexa Fluor 488 (A-11034, Thermo Fisher Scientific), Alexa Fluor™ 568 Phalloidin (A12380, Thermo Fisher Scientific).

### Promotor analyses and luciferase reporter gene assay

A 2kb region upstream of the *RANBP3L* start codon was examined through the evolutionary conserved region browser (https://ecrbrowser.dcode.org) [28]. Further analyses with the JASPAR database (http://jaspar.genereg.net) [29] showed two conserved putative NFAT5 binding sites in the region 800 bp upstream the RANBP3L start codon. A 2kb and an 800 bp fragments were amplified from C57BL/6 murine genomic DNA by PCR. Using the following primer pairs, we also introduced recognition sites for the restriction enzymes SacI and XhoI: Sequences can be found in supplemental Table 4.

The PCR products were cloned into the pGL3-MX1 (#30536, Addgene, Watertown, MA, USA) using the SacI (GAGCTC) and XhoI (CTCGAG) restriction sites. The 0.8 kb construct was used for site directed mutagenesis of the conserved NFAT5 DNA–binding sites:

GCAGTACATTTCCATGCGCTCCTGACCAGATCCAGCTGGATCT**TTTCCA**TTTCGCTT (putative NFAT5 DNA–binding sites are highlighted). Mutagenesis was performed using the QuikChange II XL Site-Directed Mutagenesis Kit, Agilent Technologies, Santa Clara, California, USA) (TabS. 4).

In total, three different mutagenesis reactions were performed. One for each NFAT5 binding site (mut 1 and mut 2) and one with both NFAT5 binding sites combined (mut 1+2). All constructs were transfected transiently in HEK293-T cells. In total, five different constructs were transfected in 96 well plate seeded cells and incubated for 24 h. After further incubation for 24h under isoosmotic (300 mosmol/kg) or hyperosmotic (450 mosmol/kg), the luciferase activity was measured with the Promega luciferase system (E1500, Promega, Madison, Wisconsin, USA) according to manufacturer’s instructions using the Infinite® 200 PRO Series Multimode Reader (Tecan Trading AG, Switzerland). Each measurement was done in biological duplicates.

### Cloning of *Ranbp3l* and generating Ranbp3l-GFP expressing cells

The cDNA of *Ranbp3l* was cloned into the pEGFP-N1 to obtain a Ranbp3l-GFP vector as described before [14]. As a control, empty pEGFP-N1 was used. These vectors were transfected in 786-0, Caki-1 and Caki-2 cells using the TurboFect™ Transfection Reagent (R0531, Thermo Fisher Scientific). For selection of positively transfected cells 0.4 mg/ml G418 (10131035, Thermo Fisher Scientific) was used.

### Conditional ex-vivo NFAT5 knockout in IMCD cells

*Nfat5*^flx/wt^-Ubc-*Cre*-ERT2^+/−^ mice were kindly provided by the group of Christoph Küper [30]*.* In these mice exon 4 of the *Nfat5* allele is flanked by *LoxP* sites. Further, these mice harbor a derivative of the *Cre*-recombinase, which is under the control of an ubiqitin-C promoter (Ubc-Cre-ERT2). The *Cre*-recombinase must be induced by a treatment with 4-hydroxytamoxifen (4-OH-TM). We further generated *Nfat5*^flx/flx^-Ubc-*Cre*-ERT2^+/-^ mice and prepared primary cultured IMCD cells [21, 22]. The cells were seeded in 24 well plates and cultivated at 300 and 600 mosmol/kg. After 48h the cells were treated for 24h with 1μg/ml 4OH-TM (T176-10MG, Sigma Aldrich), followed by additional 4 days of cultivation either at 300 or 600 mosmol/kg. The cells were further used for gene expression profiling.

### Preparation of samples for next-generation sequencing

For gene expression analysis using Next Generation Sequencing RNA-Seq, the cells were incubated for 5 days to 70-85 % confluency at 300 and 600 mosmol/kg. Total RNA was isolated using the Gen Elute Mammalian Total RNA prep kit (RTN350-1KT, Sigma Aldrich) according to manufacturer’s instructions and reverse transcribed. RNA samples from 2-4 independent separate isolations were analyzed by Novogene Co, Ltd (Cambridge, UK). The quality control, sequencing and bioinformatics were performed by Novogene as service. The detailed description can be found as supplement S1.

### Proliferation, migration and colony forming assay

For proliferation and migration assays, cells were cultivated either at 300 or 600 mosmol/kg in 96-well cell culture dishes. Data were collected with IncuCyte® Live-Cell Analysis System (Essen BioScience, Inc., Ann Arbor, MI, USA). For migration assays, the wells were grown to 100% confluency and a wound was created to the cell layer with WoundMaker™ (4493, Essen BioScience). Migration capacity was observed for 24 h and evaluated by relative wound density (RWD) using the built-in analysis software. For proliferation assays, cells were cultivated in a 96-well plate either at 300 or 600 mosmol/kg and monitored for 48 h. The doubling time was calculated by using nonlinear regression analysis and exponential growth quotation with GraphPad Prism version 8.0 (GraphPad Software Company, San Diego, CA, USA). For colony forming assay, 2,5 × 10^3^ cells, embedded in 0.2% low-melting agarose (840,006, Biozym, Hessisch Oldendorf, Germany) were plated in 48-well dishes on top of a 0.5% base agar for 2 weeks. Pictures from each well were taken by the Axio Vert.A1 FL-LED (Zeiss, Jena, Germany) with a 25x magnification and analyzed by the image j software for colony number and colony size with following settings: Substract Background: 100 pixel, Threshold: 212, Analyze Particles: 70–200, display results, summarize, add to Manager, exclude on egdes.

### Data availability

The data from the human pathology atlas allows identification of genes associated with favorable or unfavorable clinical outcome across diverse cancers. A total of 2755 favorable and 3213 unfavorable genes for renal cancer from the Human Protein Atlas Database (https://www.proteinatlas.org/humanproteome/pathology) were used [31]. Since the Human Protein Atlas does not discriminates between RCC entities we utilized the Kidney Renal Cell Carcinoma TCGA data sets for KIRC, KIRP and KICH (https://xenabrowser.net) [32]. Kaplan Meier plots and PCA were generated with GEPIA2 (http://gepia2.cancer-pku.cn/#index) [33]. Notably, GEPIA tool did not contain any data for *ADGRA2, BC025446, Siglecg,* and *H60c*. All other data of this study are available within the article or in the Supplementary files.

### Statistics

Statistical evaluation was performed using GraphPad Prism statistical software. Comparisons between two groups were analyzed using an unpaired two-tailed Student’s *t* test. For multiple group comparison one-way analysis of variance (ANOVA) was applied with a Tukey posttest. Results from bar charts are expressed as the mean ± SEM. Whiskers plots showing the 1 (lowest line) - 99 (uppest line) percentiles with a line in the box representing the median. All other values are represented as dots. The results were considered significantly different if *p* values ≤ 0.05 and is represented as follows: *, *p* < 0.05; **, *p* < 0.01; and ***, *p* < 0.001. Non-significant *p* values are shown as n.s. (p ≥ 0.05). In each graph, the number of individual experimental points are described.

## Results

### Hyperosmolality-induced *Ranbp3*l expression is triggered by NFAT5

**Fig. 1:**
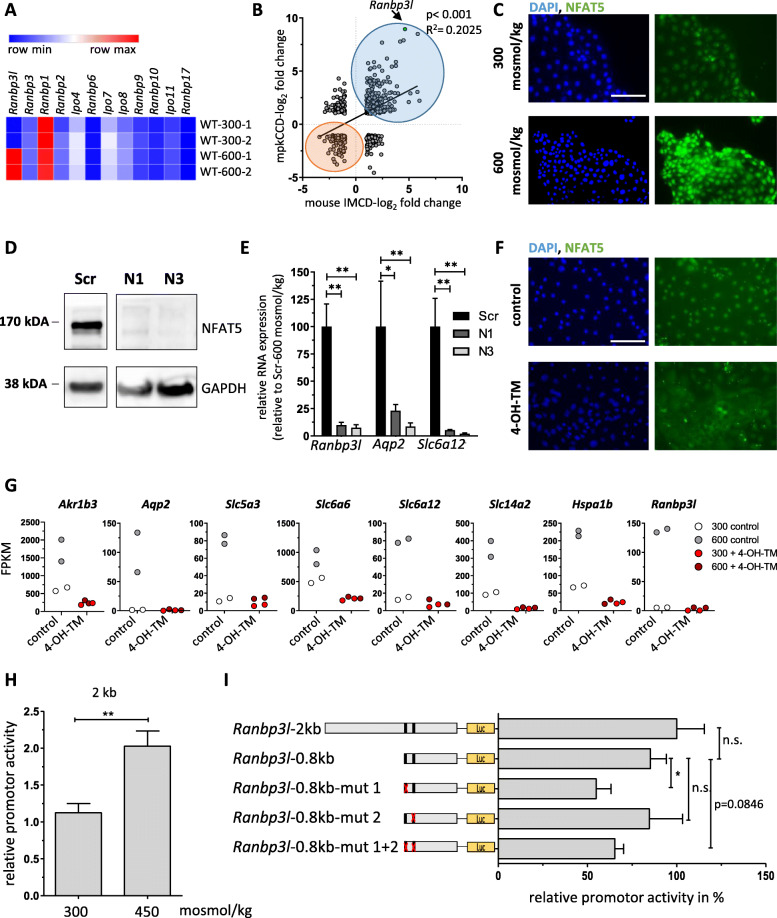
Hyperosmolality-induced upregulation of *Ranbp3l* is abrogated in NFAT5 deficient cells. **A** RNA-Seq analyses show the expression of *Ranbp3l* and other Ranbp family members in primary murine IMCD-cells cultivated at 300 or 600 msomol/kg displayed as heatmap in FPKM (Fragments per kilo base per million mapped reads), (*n* = 2). Blue to red represents lower to higher expression. **B** Pearson correlation analysis of genes affected by hyperosmolality (log_2_ fold change > 1, <− 1) between the mpkCCD cell line and primary cultivated IMCD cells. (*n =* 2–4), blue = overlapping upregulated genes, red = overlapping down regulated genes. **C** WT (wildtype) mpKCCD cells were cultivated under isosmotic and hyperosmotic conditions for 7d and subjected to immunofluorescence staining with anti-NFAT5 antibody and DAPI for nucleus visualization. Scale bar: 100 μm (**D**) Western blot analysis of NFAT5 expression in scramble (Scr) and NFAT5 deficient single cell clones N1 and N3. The expression of GAPDH served as loading control. **E** The relative expression of *Ranbp3l*, *Aqp2* and *Slc6a12* in Scr vs. NFAT5-deficient clones was analyzed by RT-qPCR in cells cultivated under hyperosmotic conditions (*n =* 3). Values represent mean ± SEM (error bars). *, *p <* 0.05; **, *p <* 0.01, 1-way ANOVA. **F** Efficient in vitro deletion of NAFT5 in primary cultured IMCD cells using *Nfat5*^flx/fl^-Ubc-*Cre*-ERT2^+/−^ mice. Representative immunofluorescence of control and 4-OH-TM treated cells for NFAT5 under hyperosmolar cell cultivation. Scale bar: 100 μm (G) FPKM values of all known kidney-specific NFAT5 targets, including *Ranbp3l* for control and 4-OH-TM treated cells at 300 mosmol/kg and 600 mosmol/kg (*n =* 2). (H) Induction of promotor activity under hyperosmotic conditions with the *Ranbp3l*-2 kb construct. (*n =* 4, each with duplicates). **, *p <* 0.01, Student’s *t* test (**I**) Luciferase assay with different *Ranbp3l* promoter fragments. Promoter activity is represented relative to the *Ranbp3l*-2 kb construct. (*n =* 4, with two technical replicates) n.s. > 0.05, *, *p* < 0.05, 1way ANOVA

RANBP3L belongs to the family of Ran binding proteins and shows a high similarity to RANBP3 (63%, TabS. 1 A, Fig. 1A). Profiling of the TCGA data set using the GEPIA2 tool (Gene Expression Profiling Interactive Analysis 2) showed ubiquitous gene expression of *RANBP3* across tissues while high expression of *RANBP3L* is restricted to the brain and kidneys, indicating a special function within these organs (Fig. 1B) [33]. This is in line with our recent finding of hyperosmolality-driven *Ranbp3l* upregulation in a rat renal cell model [14]. To assess the molecular functions of RANBP3L in more detail leveraging available mouse models for renal function and pathology, we first tested whether RANPB3L´s hyperosmolarity-driven upregulation can be translated to mice. For this purpose, we profiled the expression of Ran binding family members via RNA-Seq using primary IMCD cells isolated from C57BL/6 mice (Fig. 1C). *Ranbp3l* transcript levels were substantially elevated in the hyperosmotic setting (equivalent to 600 mosmol/kg) compared to isoosmotic conditions (equivalent to 300 mosmol/kg) (Fig. 1A). The expression of other members of the Ran binding protein family was not affected (Fig. 1A).

This hyperosmolarity-driven pattern could be reproduced in mpkCCD cells originally established from the cortical collecting duct of SV-PK/Tag mice and extensively used to study renal physiology (Fig. 1B) [16, 20, 34, 35]. Gene expression profiling in these cells showed that hyperosmolality induced extensive transcriptional changes. *Ranbp3l* was among the transcripts with the highest increase in expression. Comparison of the regulated genes between primary cultured IMCD cells and mpkCCD cell line showed a strong overall correlation of R^2^ = 0.2 (p < 0.0001) (Fig. 1B). (The complete list of differentially expressed genes is provided in Table S1).

Since NFAT5 is the key transcription factor that governs the cellular response to hyperosmolality [6, 7] we investigated its regulatory function on *Ranbp3l* expression in these cells. NFAT5 activation and nuclear translocation can be easily achieved *in-vitro* at the equivalent of 600 mosmol/kg in mpkCCD cells (Fig. 1C, profile plots of the signal intensities are shown in Fig. 1D, F) without a change in *Nfat5* mRNA expression. Therefore, we generated NFAT5-deficient mpkCCD cells by targeting two exons of the *Nfat5* gene using CRISPR/Cas9 as described before [16]. Single cell clones were isolated by serial dilution (clones N1 and N3) and successful gene disruption was validated with Sanger sequencing, TIDE (Tracking of Indels by Decomposition) analyses and TOPO-TA cloning (Fig. 1H, TabS. 2 A). As a control we used a non-targeting scrambled oligo (Scr) as described before [16]. Immunoblotting confirmed the loss of NFAT5 protein in both clones (Fig. 1D). Next, we analyzed the effect of the NFAT5 deletion on known NFAT5 target genes by RT-qPCR. As compared to Scr-control , the expression of *Aqp2 and Slc6a12,* two common NFAT5 target genes [35, 36] and *Ranbp3l* was significantly decreased in NFAT5-KO cells under hyperosmotic conditions. (Fig. 1E). These results indicate that NTFA5 is involved in the hyperosmolar induced expression of *Ranbp3l* in mpkCCD cells.

To further validate these findings, we applied an inducible *NFAT5* knockout mouse model as described by Küper *et al*. [30]. The CRE-mediated deletion of the *NFAT5* exon 4 was induced *in vitro* by incubating the cells with 4-OH-TM (4-Hydroxytamoxifen) for 24h followed by further incubation of the cells for 5 days. Successful deletion of the floxed *Nfat5* exon was confirmed by a loss of nuclear NFAT5 protein (Fig. 1F, profile plots of the signal intensities are shown in Fig. 1E, G). Quantification of transcripts by RNA-Seq showed that under hyperosmotic conditions *Ranbp3l* as well as classical NFAT5 target genes like *Akr1b3, Aqp2, Slc5a3, Slc6a6, Slc6a12, Slc12a2* and *Hspa1b* were not up regulated as compared to 4-OH-TM untreated cells (Fig. 1G, TabS. 2B-C) thus corroborating the results from the mpkCDD cells.

Next, we asked whether the NFAT5-dependent expression of *Ranbp3l* is mediated by a direct NFAT5-RANBP3L promoter interaction. By *in-silico* analyses, we found two conserved putative NFAT5 binding sites within the 2 kb region upstream of the *Ranbp3l* transcription start site (Fig. 1K). To test the binding capacity of these two sites, we generated luciferase reporter constructs encompassing the first 2 or 0.8 kb of the *Ranbp3l* promoter region (both fragments contain all putative NFAT5 DNA–binding sites, Fig. 1L). For the 2 kb fragment, luciferase activity was induced by hyperosmolality (Fig. 1H). In addition, we introduced mutations in either one or both predicted NFAT5 DNA–binding sites. Relative luciferase activity was measured between cells cultivated at 300 mosmol/kg and 450 mosmol/kg. As shown in Fig. 1I, there was no considerable difference in activity between the 2kb and 0.8kb constructs under hyperosmotic conditions, indicating that the essential regulatory information needed to drive *Ranbp3l* transcription is encoded within the 0.8 kb region. In contrast, 50-60% of the hyperosmolality-induced luciferase activity was lost in all reporter constructs harboring a mutation within the putative NFAT5 binding site 1 (Fig. 1I, L). Mutation in the potential NFAT5 binding site 2 did not result in a decreased promotor activity, which lead to the hypothesis that this site is not functional in the applied assay (Fig 1I). Notably, site 1 is more conserved across species as compared to site 2 (Fig. 1J, K). In summary, *Ranbp3l* expression is directly triggered by NFAT5.

### RNBP3L deficiency changes the morphological structure of murine cortical collecting duct cells

**Fig. 2:**
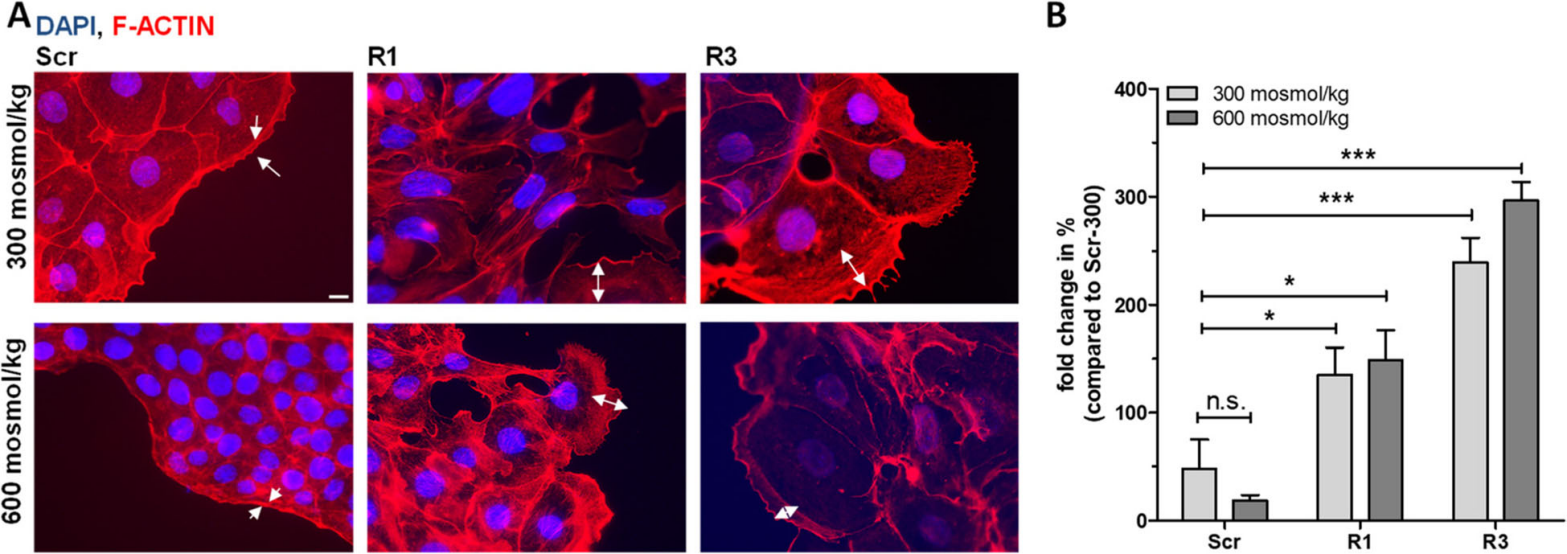
Loss of RANBP3L induces morphological changes in mpKCCD cells. **A** Staining of the F-actin with Alexa-568 coupled phalloidin in Scr, R1 and R3 mpkCCD cells, staining of nucleus with DAPI (Scale bar: 10 μm). White arrows indicate lamelopodia boundary. **B** Fold change of area covered by lamelopodia of Scr cells compared to RANBP3L-deficient cells. Area covered by lamelopodia was measured with the Zen-blue software. For each condition 2–4 cells from 3 independent biological replicates were measured (*n =* 3). Values represent mean ± SEM (error bars). n.s., *p* > 0.05, *, *p <* 0.05, ***, *p <* 0,001, 1-way ANOVA

The cellular function of RANBP3L in renal cells has not been described so far. For initial characterization of RANBP3L we used CRISPR/Cas9 to generate RANBP3L-deficient mpkCCD cells as described above (Fig. 2C, D, TabS. 3 A). Two different single cell clones (R1 and R3) from two different targeting gRNAs were used for further characterization. Loss of basal *Ranbp3l* expression cells alter cellular morphology and led to partial epithelial disassembly (Fig. 2A).

While Scr-control cells showed a typical „cobblestone“ epithelial morphology with a compact, round-shaped structure and less contact between the cell clusters, the RANBP3L-KO cells exhibited a fibroblast-like morphology with reduced cell-cell contacts (Fig. 2A, B). Labeling of the F-Actin filaments showed actin enrichment predominantly at the cell-cell contacts in Scr cells, indicating an intact epithelial structure and apical-basal polarity. In contrast, RANBP3L-KO cells lost their epithelial structure. This effect was even stronger in cells cultured under hyperosmotic conditions (Fig. 2A, B). The RANBP3L-KO cells also formed cellular protrusions similar to lamellipodia. The covered area of the protrusions was nearly two-three-fold bigger in RANBP3L-KO cells at 300 momsol/kg and six-eight-fold bigger at 600 momsol/kg compared to Scr cells (Fig 2B). Collectively, our results demonstrate, that RANBP3L deficiency changes the morphological structure of murine cortical collecting duct cells.

### Loss of RANBP3L induces cellular transformation towards a tumor like phenotype

**Fig. 3:**
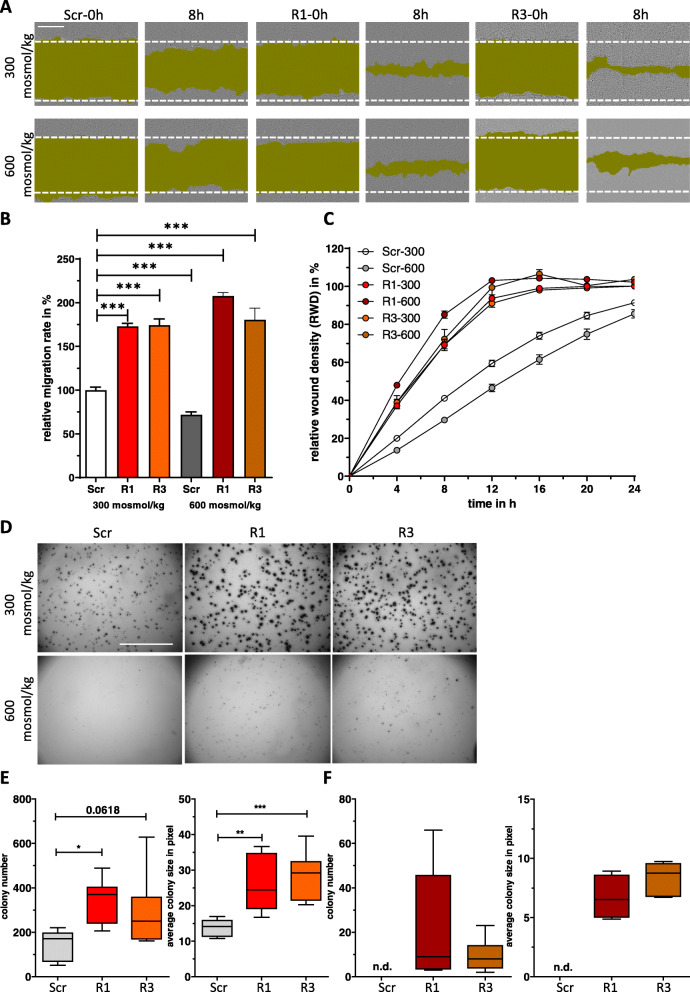
RANBP3L deficiency induces cell migration and colony forming capacity. **A** Representative images of the migration assay. Scr**,** R1 and R3 mpkCCD cells were cultivated in 96 well plates and cell migration was monitored by live cell imaging using IncuCyte S3 system. The pictures show the progressive closure of an induced wound at indicated time points (0 h and 8 h). Wound areas are labeled in dark green. Scale bar: 400 μm. **B** Plot of the wound density over time. Migration assay was performed for 24 h. Values represent mean ± SEM (error bars). *n =* 6 (with 4 technical replicates). **C** The relative migration change after 8 h was calculated by linear regression analysis using GraphPad Prism. ***, *p <* 0.001, 1-way ANOVA. **D** Representative images of Scr, R1 and R3 cells during colony forming assay. After 2 weeks of incubation 4–5 images per well were taken and stacked with the image J software tool. Scale bar: 500 pixels. **E-F** Colony number and colony size at 300 mosmol/kg (**E**) and 600 mosmol/kg (**F**). Values are represented in a whiskers 1–99 percentile plot. n.d. = not detectable, *, *p <* 0.05, **, *p <* 0.01, ***, *p <* 0.001, 1-way ANOVA

Since RANBP3L-KO cells showed morphological changes towards a fibroblast-like phenotype, which represents a hallmark of tumor cell migration [37], we analyzed if loss of *Ranbp3l* expression also affects functional parameters like cell migration, proliferation and colony forming capability. By wound healing assays, we showed a significant higher migration rate of RANBP3L-KO cells compared to Scr-control cells (Fig 3A-C). Proliferation assays and determination of mean cell doubling times excluded cell division as driving force of these effects (Fig. 2E, F). Hyperosmolality had a negative impact on cell migration capability in Scr-cells. This effect was not evident in RANBP3L-deficient cells.

Next, we assessed anchorage-independent growth of RANBP3L-KO cells by soft agar colony formation assay. Under isoosmotic conditions, the RANBP3L-deficient cell clones exhibited significant higher colony forming ability with bigger colonies as compared to the Scr cells (Fig. 3D-F). Interestingly, hypersomolarity completely abrogated anchorage-independent growth of Scr cells, while RANBP3L-deficient cells maintain low-level colony formation capabilities (Fig. 3D-F). Together, loss of RANBP3L induces transformation towards a tumor cell-like phenotype.

### Loss of RANBP3L correlates with a gene signature unfavorable for RCC

**Fig. 4:**
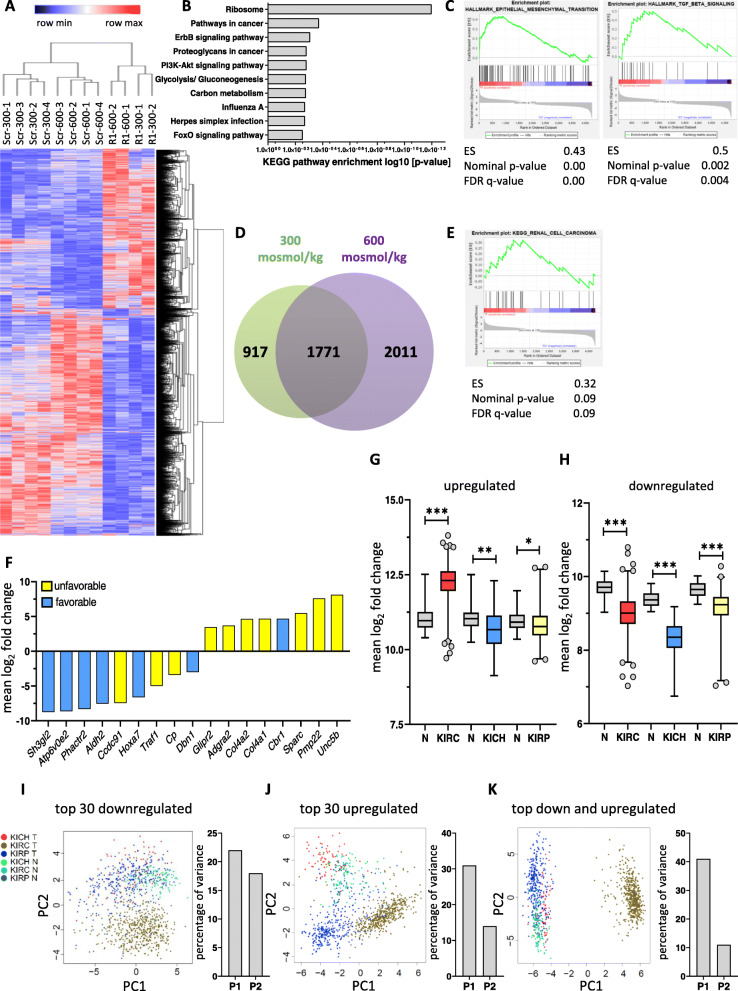
Loss of Ranbp3l induces massive changes in gene expression representative of clinical RCC. **A** Hierarchical clustering of altered samples based on the FPKM values. Cluster analysis was performed using all significant regulated genes in R1 cells. Blue to red represents lower to higher expression. **B** The list of differentially expressed genes between Scr and R1 mpkCCD cells cultivated under 300 and under 600 mosmol/kg were analyzed for enrichment of KEGG pathways. The top 10 enriched pathways are presented here. (**C**) Gene set enrichment analysis (GSEA) of RNA-Seq results shows induction of EMT and TGF-β pathways. **D** Venn diagram showing the overlap of 1771 genes with equally altered gene expression at 300 and 600 mosmol/kg in R1 cell clones. **E** GSEA of deregulated genes showing an enrichment within the KEGG Renal Cell Carcinoma pathway. **F** List of regulated genes with a log_2_ fold change of 3 or higher − 3 or lower and a FPKM value higher than 10 were compared with genes that have a prognostic impact on patient’s outcome with renal cell carcinomas (RCC) from the human pathology atlas. Unfavorable genes are indicated in yellow while favorable genes are blue. (**G**, **H**) Mean log_2_ gene signature expression of top upregulated (not including *ADGRA2*) (**G**) and top downregulated RCC biomarkers (H) in RCC subtypes KIRC (red), KICH (blue) and KIRP (yellow) samples compared to normal tissue (N, grey). Values were analyzed using a student’s-T test and are represented in a whiskers 1–99 percentile plot, n.s. > 0.05, *, *p <* 0.05, **, *p <* 0.01, ***, *p <* 0.001.1-way ANOVA. Data was obtained from the TCGA database [32]. **I-K** Principal component (PC) analyses and the corresponding percentage of variances of the top 30 downregulated (**I**), top 30 upregulated (**J**) genes and both gene sets together (**K**)

Based on the morphological and functional differences observed between Scr and RANBP3L-KO cells we hypothesized that these alterations are associated with changes in the cellular gene expression profile. To identify signaling pathways mediating the phenotypical alterations, we performed RNA-Seq using Scr and R1 cells cultured at 300 and 600 mosmol/kg. Statistical analysis revealed a total of 4699 significantly deregulated genes in RANBP3L-deficient cells as compared to the Scr-control (Table 1, TabS. 3 B, C). Hierarchical clustering clearly separated the Scr cells from RANBP3L-KO cells and within these groups, the samples clustered according to their cell culture conditions (Fig. 4A). In *in-silico* of KEGG (Kyoto Encyclopedia of Genes and Genomes) analysis of the 4699 differentially expressed genes (DEGs) revealed an enrichment of pathways associated with cancer (Pathways in Cancer, Proteoglycans in cancer and PI3K-Akt pathway) (Fig. 4B). These results were confirmed by gene set enrichment analysis (GSEA) showing an enrichment of EMT **(**Epithelial-to-mesenchymal transition) and TGF-ß associated pathways (Fig. 4C, Fig. S3 A-B). Further dissection of the 4699 DEGs uncovered deregulation of 1771 genes, overlapping in iso- and hyperosmotic conditions. In contrast 917 genes were exclusively altered at isotonic conditions, while 2011 genes were differentially expressed under hyperosmotic conditions only (Fig. 4D). Moreover, GSEA showed the tendency for an enrichment of genes from the KEGG Renal Cell Carcinoma gene set in RANBP3L-KO cells (Fig. 4E).

**Table 1:** Top 30 up and downregulated genes in RANBP3L-deficient cells. Genes deregulated under isosmotic and hyperosmotic cell culture conditions are highlighted in bold

Top 30 upregulated genes				Top 30 downregulated genes			
300 mosmol/kg		600 mosmol/kg		300 mosmol/kg		600 mosmol/kg	
Gene	log_2_fold change	Gene	log_2_fold change	Gene	log_2_fold change	Gene	log_2_fold change
***Pmp22***	8.10	***Unc5b***	8.88	***Hoxa9***	−10.23	***Zfp422***	−9.10
***Unc5b***	7.56	**Pmp22**	7.65	***Hoxa10***	−9.91	*Plagl1*	−8.81
***Tmem30b***	7.35	**Syt17**	7.532	***Epdr1***	−9.39	***Sh3gl2***	−8.71
***Syt17***	6.84	**Tmem30b**	7.41	***Hdgfrp3***	−9.31	***Hoxa10***	−8.59
***BC025446***	6.19	**Ica1**	6.63	***Zfp422***	−9.28	***Asb4***	−8.44
***Sparc***	6.08	***BC025446***	6.55	***Atp6v0e2***	−8.80	***Atp6v0e2***	−8.44
***Gnao1***	5.85	***Siglecg***	6.47	***Sh3gl2***	−8.77	***Galnt11***	−8.38
***Ica1***	5.84	***Gnao1***	5.86	***Phactr2***	−8.64	*Arrb1*	−8.31
*Lrrc32*	5.66	***Sparc***	5.08	***Eif5a2***	−8.33	***Hoxa9***	−8.14
***Gcnt****3*	5.55	***Gcnt3***	5.08	***Galnt11***	−7.95	***Smoc1***	−8.13
***Acp5***	5.51	*Parvb*	4.96	***Inmt***	−7.84	***Phactr2***	−7.98
***Col4a1***	5.35	***Parm1***	4.91	***Smoc1***	−7.73	***Inmt***	−7.86
***Gstk1***	5.32	*Cntfr*	4.85	*Rnf217*	−7.72	***Epdr1***	−7.80
***Col4a2***	5.20	***Cbr1***	4.84	***Hebp2***	−7.66	***Aldh2***	−7.57
***Col4a3***	5.15	***Acp5***	4.82	***Aldh2***	−7.49	***Hdgfrp3***	−7.57
***Parm1***	5.03	***Col4a3***	4.80	***Ccdc91***	−7.48	***Ccdc91***	−7.39
***Siglecg***	4.89	*Parvg*	4.77	***Hoxd9***	−7.46	***Hebp2***	−7.36
*Itgb7*	4.71	*Sytl5*	4.70	*Hoxd8*	−7.29	*Neil3*	−7.20
*Gc*	4.64	*Mcpt8*	4.63	***Slc9a7***	−7.26	***Slc9a7***	−7.14
*Parvb*	4.63	***Gstk1***	4.34	***Sim1***	−7.25	*Adgra1*	−7.08
*Dmkn*	4.62	*Sftpd*	4.24	***Sipa1l2***	−7.15	***Sim1***	−7.04
***Cbr1***	4.59	***Myo7b***	4.22	*Bckdhb*	−7.05	*Zfhx4*	−7.03
***Myo7b***	4.55	*Serpinb9b*	4.16	***Tspan13***	−7.02	*Nrk*	−7.00
*Lrrc61*	4.42	*Dhrs9*	4.16	***Enox2***	−6.96	***Enox2***	−6.99
*Col4a4*	4.36	*Nid1*	4.14	***Asb4***	−6.95	*Kitl*	−6.90
*Bgn*	4.34	***Col4a2***	4.13	*Hoxd10*	−6.73	***Hoxd9***	−6.85
*Trex2*	4.28	*Ociad2*	4.11	*Prdm5*	−6.68	***Tspan13***	−6.77
*Inhba*	4.18	*C130073F10Rik*	4.07	*Pdlim1*	−6.68	***Eif5a2***	−6.75
*H60c*	4.13	***Col4a1***	4.05	*Nrk*	−6.66	***Hoxa7***	−6.64
*Glipr2*	4.12	*Lypd5*	4.03	***Hoxa7***	−6.62	***Sipa1l2***	−6.61

To link the RANBP3L dependent gene expression profile to human RCC, we compared the 1771 osmo-independent DEGs to a RCC gene set from the human pathology atlas (Fig. 3C), listing genes that correlates with a favorable or unfavorable clinical outcome (TabS. 3D). Overall, we found 290 favorable and 333 unfavorable matched genes (Fig. 3C).

Interestingly, favorable and unfavorable genes were equally distributed among the group of genes downregulated in the *RANBP3L*-deficient cells (18 % and 17%, respectively). In contrast, a higher number of unfavorable genes (23%) was induced in *RANBP3L*-KO cells as compared to favorable genes (11%). Notably, we confirmed up- and downregulation of selected matched genes via RT-qPCR (Fig. 3D, E). We further confined the set of 623 prognostic matched genes, by extracting only those with a log_2_ fold change of at least >3 or ≤-3 and a mean FPKM value higher 10 to include only highly differentially altered transcripts (Fig. 4F, Table 2). The majority of the 17 remaining genes was associated with an overall unfavorable prognosis in RCC (Fig. 4F). To conclude, our data suggest that loss of RANBP3L results in an unfavorable gene signature for RCC.

**Table 2:** Mean FPKM values of the top down- and upregulated prognostic genes for RCC

	FPKM-Scr-300 mosmol/kg	FPKM-R1–300 mosmol/kg	FPKM-Scr-600 mosmol/kg	FPKM-R1–600 mosmol/kg
**downregulated**				
*Sh3gl2*	11.35	0.025	10.44	0.024
*Atp6v0e2*	15.92	0.035	17.78	0.05
*Phactr2*	11.49	0.028	26.09	0.10
*Aldh2*	26.50	0.146	23.55	0.12
*Ccdc91*	14.31	0.079	17.15	0.10
*Hoxa7*	24.43	0.24	23.82	0.23
*Traf1*	30.92	0.90	22.92	0.80
*Cp*	25.05	3.38	82.04	5.39
*Dbn1*	16.05	2.27	13.90	1.50
**upregulated**				
*Unc5b*	0.06	11.39	0.02	11.75
*Pmp22*	0.04	12.64	0.05	11.16
*Sparc*	1.37	93.42	2.60	88.70
*Cbr1*	0.70	16.99	0.67	19.37
*Col4a1*	3.17	130.11	6.42	106.96
*Col4a2*	3.89	144.28	6.54	115.17
*Adgra2*	1.05	14.99	0.91	11.37
*Glipr2*	1.13	19.71	2.73	23.68

Since the RNA-Seq of RANBP3L-deficient cells revealed a significant impact on genes prognostic for RCC, we analyzed the correlation of RCC subtypes and the gene signatures of the 8 upregulated or 9 downregulated genes, dissected by our stringent sorting (Fig. 4F). As compared to normal tissue, the set of upregulated genes was especially induced in the KIRC cohort (Fig. 4G), while levels of the downregulated gene set were equally decreased in all three RCC subtypes (KIRC, KICH and KIRP) (Fig. 4H). Further, correlation analyses of *RANBP3L*-dependent gene expression with the up- and downregulated signatures in the KIRC TCGA cohort (Fig. 3F-G) revealed a strong association between the RANBP3L-KO signature in mpKCCD and a KIRC-like profile.

We next tested whether the gene signature detected in the RANBP3L-KO cells matches the expression profiles of the human KIRC, KIRP, and KICH samples from the TGCA RCC cohort using principal component analysis (PCA). For PCA, we compared the expression of the top 30 up- and downregulated genes from our RNA-Seq analysis (Table 1) with their levels in the respective RCC subtype. Visualization of the first two principal components revealed a subtype-specific clustering for the top 30 upregulated genes (Fig. 4I-K), while the top 30 downregulated genes were more specific for KIRC (Fig. 4I). Notably, combination of up- and downregulated signatures clearly separated KIRC samples from the rest (Fig. 4K). In summary, loss of RANBP3L is associated with a gene signature unfavorable for KIRC.

### *RANBP3L* is downregulated in RCC and affects prognosis in the KIRC subtype

**Fig. 5:**
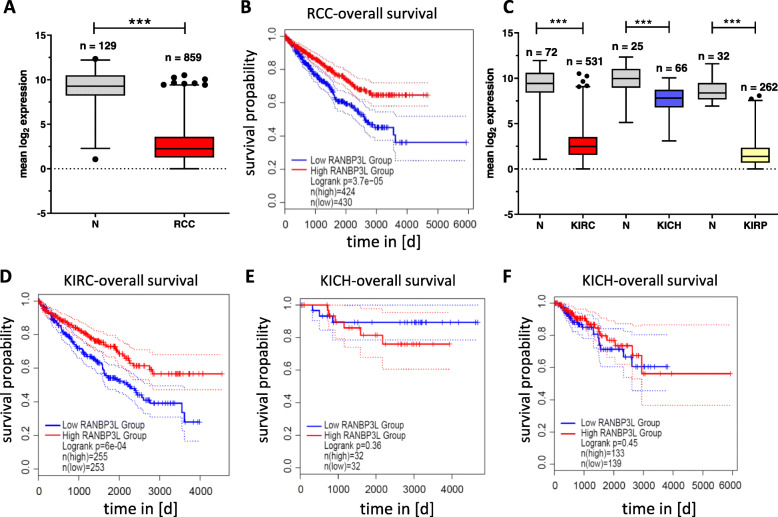
*RANBP3L* is downregulated in RCC cohorts and has a KIRC specific prognostic value. **A** Mean log_2_ expression of *RANBP3L* in all TCGA RCC samples compared to normal tissue. Values were analyzed with a student’s-T test and are presented in a whiskers 1–99 percentile plot, ***, *p<*0.001. *n =* the number of available samples in the indicating TCGA cohort. **B** Kaplan Maier Plot of *RANBP3L* for RCC (all cohorts). **C** Among RCC subtypes *RANBP3L* expression is downregulated in KIRC, KICH and KIRP. Values were analyzed with a student’s-T test and are represented in a whiskers 1–99 percentile plot, ***, *p<*0.001. *n =* the number of available samples obtained from TCGA [32]. **D-F** Kaplan Maier Plot for *RANBP3L* in the different RCC subtypes KIRC (**D**). KICH (**E**) KIRP (**F**). All Kaplan Maier Plots were generated using GEPIA2 tool [33] with the corresponding RCC cohort of TCGA data and solid tissue normal samples (N) (high): Samples with expression level higher than the median are considered as the high-expression cohort. N (low): Samples with expression level lower than the median are considered the low-expression cohort. Logrank test

Since we could link the deficiency of murine *RANBP3L* to the expression of prognostic factors for RCC (Fig. 4F), we next analyzed *RANBP3L* expression in the TCGA RCC samples. In general, *RANBP3L* is extensively downregulated in RCC as compared to normal tissue (Fig. 5A). Furthermore, a high *RANBP3L* expression positively correlated with a longer overall survival in general RCC (Fig. 5B). Although downregulation of *RANBP3L* was independent of the KIRC, KICH and KIRP subtypes (Fig. 5C), the downregulation was more pronounced in KIRC and KIRP than in KICH (Fig. 5C). However, only in the KIRC subtype high *RANBP3L* expression exhibits a positive correlation with survival, while *RANBP3L* expression does not affect patient outcome in the KICH and KIRP entities (Fig. 5D-F). Considering that *RANBP3L* has a prognostic value exclusively for KIRC patients’ overall survival we further analyzed *RANBP3L* expression in KIRC cell lines 786-0, Caki-1 and Caki-2 cell. *RANBP3L* mRNA expression was only evident under hyperosmotic cell culture conditions, which also correlated with NFAT5 nuclear translocation (Fig. S6). To get more insights in RANBP3L functional role without hyperosmolar sight effects we transfected 786-0, Caki-1 and Caki-2 with a Ranbp3l-GFP vector and identified differences in cell proliferation and migration (Fig. S7). We could see in all cell lines that RANBP3L overexpression leads to a reduction of migration (Fig. S7). Proliferation rate and doubling time was also strongly affected by a RANBP3L-GFP vector for Caki-1 and Caki-2 (Fig. S7). Lastly, our results indicate that besides RANBP3L´s prognostic role there is also a functional meaning in KIRC.

## Discussion

The tumor microenvironment plays a critical role in cancer progression [38]. This includes soluble factors [39], pH [40] but also the environmental osmolality [41]. We have recently shown that a the hyperosmotic environment, is capable to affect the expression of more than 100 prognostic genes in KIRC and that genes or factors associated with renal osmo-homeostasis are deregulated [15, 16], including *RANBP3L* [14, 15].

Since RANBP3L might represent a novel target for the treatment of KIRC patients, knowledge about the regulation of its expression is of major importance. Recently, we identified hyperosmolarity as one factor that induces *Ranbp3l* expression in rat IMCD cells [14]. In this study, we extended this finding by establishing *Ranbp3l* as novel NFAT5 target gene under hyperosmotic conditions using NFAT5-deficient mpkCCD and primary cultivated IMCD cells. The NFAT5-deficient IMCD cells we used in our experiments shared common features of NFAT5-mediated osmoregulation reported for transgenic mice and embryonic fibroblasts that lacked NFAT5 activity [42, 43]. Using this model we could show, that loss of NFAT5 reduces hyperosmotic *RANBP3L* expression and renders canonical osmolality genes like *Aqp2*, *Slc5a3*, *Slc6a6* or *Slc6a12* [35, 42, 43], less or non-responsive, a correlation that has also been observed for KIRC specimen [8]. Further, *NFAT5* itself is strongly reduced in RCC. By looking at the entities this effect is the strongest in the KIRC subtype (Fig. S5). A main aspect of KIRC development is the mutation of VHL [2]. Since studies already describe a VHL dependent increase in medullary vascularization (IMV) resulting in hypoosmotic urine [5] we further could show that a VHL-KO in a normal murine kidney cell line inferred also with a hyperosmotic and prognostic unfavorable gene expression [16]. However, VHL-dependent regulation of NFAT5 remained unclear. Our data so far indicates at least an interaction of VHL with *NFAT5* and *RANBP3*L expression and further analysis are needed to identify the molecular mechanisms in more detail. In cancer, multifaceted roles have been reported for NFAT5. For example, NFAT5 promotes T cell proliferation and activation in thymoma [13] and stimulates invasion of melanoma cells [10]. In contrast, NFAT5 promotes apoptosis by inhibiting cell cycle progression in hepatocellular carcinoma [12] and is targeted by microRNAs in RCC which results in poor survival of patients [8]. Since VHL mutation alone did not induce renal cell carcinoma [3] in mice model it might be interesting to test if a VHL and NFAT5 double KO mouse would develop a renal cell carcinoma.

A mechanistical explanation for RANPB3L´s prognostic value in RCC is provided by the observation, that the loss of its expression in mpkCCD cells induces a cellular transformation towards a tumor like phenotype including loss of epithelial structure, higher migration and colony forming capacity all of which are key hallmarks of carcinogenesis [37, 44]. **Besides functional differences, loss of RANBP3L is accompanied by a massive shift towards a gene expression profile that is linked to different cancer-associated pathways including the phosphatidylinositol 3 kinase (PI3K)/Akt, EMT or TGF-β signaling cascades. This is in line with RCC studies reporting highly active** PI3K/Akt [45] and, consistent with our results of faster migration, TGF-β induced EMT [46]. **Further** our data is supported by the observation that the nuclear export of Smads by RANBP3L regulates bone morphogenetic proteins (BMP) belonging to the TGF-ß superfamily [19, 47].

In our RNA-Seq data we could find 42 % of the differentially expressed genes under hyperosmolality while 19 % were deregulated only under isosmotic condition supporting our hypothesis that RANBP3L has a more pronounced function in tissues dealing with high extracellular osmolality like the kidney. From this, we suggest that loss of RANBP3L could lead to specific dysfunctions and diseases. In the study presented here, we assessed *RANBP3L* expression in general RCC as well as in the KIRC, KICH and KIRP subtypes. Although all RCC subtypes share low levels of *RANBP3L*, higher expression positively correlates with overall survival in KIRC but not in KICH and KIRP suggesting *RANPB3L* downregulation as major KIRC-specific feature during cancer progression. Mining of the TCGA mutation database did not reveal widespread mutations or losses within the genomic region encompassing the *RANBP3L* gene (Fig. S4 A-C) that could explain the described transcription levels thus arguing for actual downregulation of *RANBP3L* in RCC.

Furthermore, the comparison of our data with data from the Human Pathology Atlas [31] showed that, RANBP3L deficiency predominantly induced the expression of genes that are associated with an unfavorable outcome of patients with RCC. Strikingly, a RANBP3L dependent gene signature clearly separated the KIRC cohort from the other RCC subtypes and normal tissue control. To our knowledge we are the first describing a gene signature, that is mediated by loss of function for a specific gene, that clearly separates KIRC from other RCC entities and normal tissue. Known studies related their gene signature to the survival of RCC patients [48–51] or to metastatic potential of the tumor [52], which together could work as a tool for novel RCC therapeutic decisions. Although we used a murine instead of human cell lines, our data, together with our recently published study using primary rat IMCD cells [15], suggests a translational value for KIRC patients. Another potential limitation is the use of cells originating from the collecting duct [34], because in the traditional view KIRC arises from cells of the proximal tubulus [53]. However, there is also evidence that KIRC subsets can originate from the distal tubulus or collecting duct cells [54, 55]. Our KIRC expression profile is also congruent to data from Frew *et al.* who generated a KIRC-like mouse model via simultaneous knockout of VHL, TRP53 and RB1 [56]. In this model, *Ranbp3l* is downregulated (Fig. 4D) and newly identified RANBP3L dependent genes like *Sparc*, *Col4a1* and *Col4a2* are upregulated, a feature already reported for RCC development and progression [57, 58]. Interestingly, SPARC was described as key mediator of TGF-β-induced metastasis in RCC cell lines [57] corroborating the morphological and phenotypical changes we observed in RANBP3L-deficient cells. Further, the ectopic expression of *Ranbp3l* in the KIRC cell lines 786-0, Caki-1 and Caki-2 attenuated their cell migration behavior and reduced the cell proliferation in Caki-1 and Caki-2. Xenograft based studies of RANBP3L-KO mpkCCD cells and KIRC cell lines would give us more information regarding cancer growth and metastasis. Together our results indicate that RANBP3L has not only a prognostic value for overall survival of KIRC patients but is also crucial for development and progression of KIRC.

Besides KIRC, KICH and KIRP, the renal collecting duct cell carcinoma (CDC; also known as Bellini duct carcinoma) represents another rare (less than 2%) and aggressive RCC entity [59]. There are only limited reports available for CDC but gene expression profiling showed that a CDC-specific gene set clustered with KIRC [60, 61] and that a strong downregulation of *RANBP3L* is characteristic for CDC samples as compared to the control group (Fig. 4E, F) [61, 62].

**Fig. 6:**
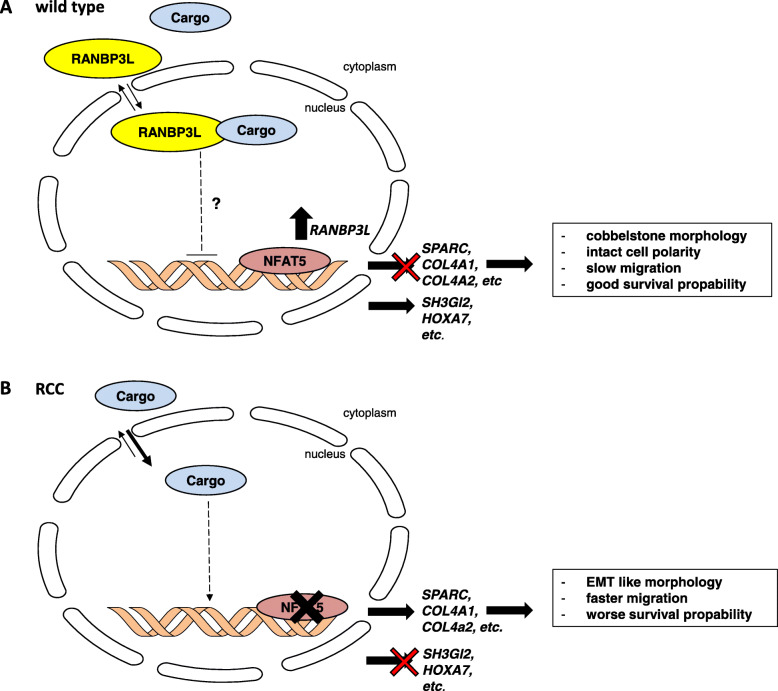
Schematic summary of RANBP3L characterization. Schematic representation of WT (**A**) and RCC cells (**B**) with downstream effectors responsible for phenotypical behavior

However, the information regarding cellular functions of RANBP3L is limited and a mouse model lacking *Ranbp3l* expression is not available. Since RANBP3 was classified as a TGF-β terminating protein by translocating cargo proteins from the nucleus back to the cytoplasm [62], we assume a similar role for RANBP3L in the kidney. Therefore, further analysis of cellular functions of RANBP3L and the identification of its cargo proteins could help to understand, how loss of function can induce such a huge change in cellular phenotype. In addition, further experiments revealing the downstream signaling of RANBP3L are needed. Figure 6 summarizes our postulated role of RANBP3L.

## Conclusion

In conclusion, we could show that the osmoregulatory NFAT5 directly drives *Ranbp3l* expression and that loss of RANBP3L leads to a cancer promoting phenotype associated with human RCC, predominantly the KIRC subtype. The transcriptome of RANBP3L-deficient cells is characterized by upregulation of important effectors of key signaling pathways, which could serve as new therapeutic targets in RCC as RANPB3L itself.

## Supplementary Information

**Additional file 1: Word file S1**. The detailed description of quality control, sequencing and bioinformatics of Novogene.

**Additional file 2: Table S1**. AS sequence of RANBP3L and RNA seq data from murine IMCD cells at 300 and 600 mosmol/kg

**Additional file 3: Table S2**. CRISPR Cas 9 target sites and TOPO TA results for NFAT5 Knockout cells and mouse NFAT5-KO RNA-Seq at 300 and 600 mosmol/kg

**Additional file 4: Table S3**. CRISPR Cas 9 target sites and TOPO TA results for RANBP3L Knockout cells, prognostic RCC genes from Human Protein Atlas and TCGA RCC data sets

**Additional file 5: Table S4**. Complete primer list.

**Additional file 6: Figure S1.** (A) Schematic representation of domain organization in RANBP3L and RANBP3 (AS sequence in Table S1). (B) Median gene expression of RANBP3 and RANBP3L in a bodymap from the gene expression profiling interactive analysis tool (GEPIA2) [33]. (C) Schematic representation of IMCD isolation and culturing from murine kidneys for next-generation sequencing (NGS). (D-E) Analysis of NFAT5 intensity in mpKCCD immunofluorescence images (D) and in IMCD cell immunofluorescence images (E) shown in Fig. 2A. Scale bar 100 μm. (F-G) The NFAT5 fluorescence intensities of cells were plotted on a line graph. Lines correspond to the relative fluorescence of cells marked with a white arrow. F corresponds to signal intensity in mpkCCD cell while G corresponds to signal intensity of IMCD cells. (H) TIDE analysis of N1 and N3 single clones reveal INDELs in the *Nfat5* target sites [26]. (I) Selected distribution of RNA-Seq reads across the *Nfat5* gene for control and 4-OH-TM treated cells shows loss of exon 4. (J) Screen capture of human *RANBP3L* promotor region from the ECR Browser website with evolutionary conserved regions (ECR) in the genomes of rat, mouse, canis, macaca and pan. The lines in pink (arrows ahead) show ECR between human and the indicated mammals [28]. (K) 800 bp region uptream from *RANBP3L* start site is conserved in the indicated mammals and contains one conserved NFAT5 consensus sequence (NNTTTCCA is indicated in yellow) and one non-conserved (nucleotides are indicated in red). (L) Relative promotor activity of all *Ranbp3l* promotor constructs normalized to the isoosmolar control (*n* = 4, each with duplicates). n.s. > 0.05, *, *p* < 0.05, **, *p* < 0.01. Student’s *t* test.

**Additional file 7: Figure S2.** (A) Phase contrast images of Scr, R1 and R3 mpkCCD cells with 300 mosmol/kg and 600 mosmol/kg. Arrowheads indicate colony-detaching cells. Scale bar: 400 μm (B) Fluorescence of F-Actin staining in Scr, R1 and R3 mpkCCD cells using Phalloidin coupled to Alexa-568 and DAPI for nucleus visualization (Scale bar: 100 μm). (C-D) TIDE (Tracking of Indels by Decomposition) analysis of R1 (C) and R3 (D) single clone revealing INDELs in *Ranbp3l* target site [26]. (E-F) Proliferation analyses of Scr, R1 and R3 mpkCCD cells. Cells were cultivated in 96-well plates and the proliferation was measured by live-cell imaging using the IncuCyte S3 system taking an image every 4 h for 24 h. The relative cell density was calculated by IncuCyte S3 software and normalized to the cell density at 0 h. The data points were fitted by nonlinear exponential growth equation using GraphPad Prism to calculate the mean doubling times. Values represent mean ± SEM (error bars). *n* = 3 (with 4 technical replicates) n.s.,*p* > 0.05, **, *p <* 0.01; ***, *p <* 0.001, 1-way ANOVA.

**Additional file 8: Figure S3.** (A-B) GSEA of RANBP3L dependent genes shows enrichment of the REACTOME Paythways “degradation of extracellular matrix” and “collagen formation” in RANBP3L-KO cells. (C) Mean log_2_ fold change of 1771 overlapping genes with prognostic favorable (blue), unfavorable (yellow) and uncategorized (black) genes in RCC. (D-E) RT-qPCR validation of different RANBP3L-KO clones (each in triplicates) for (D) *Sparc*, *Col4a1*, *Col4a2* and (E) *Sh3gl2*, *Aldh2* and *Hoxa7*. Values represent mean ± SEM (error bars), n.s. > 0.05, *, *p <* 0.05, **, *p <* 0.01, Student’s *t* test. (F-G) Pearson correlation analyses of *RANBP3L* expression and the upregulated (F, not including *ADGRA2*) and downregulated (G) signature expression in KIRC using the TCGA data set.

**Additional file 9: Figure S4.** (A-C) Somatic mutation analysis of *RANBP3L* in KIRC (A), KICH (B) and KIRP (C). (D) RNA-Seq analysis of WT (wildtype) and Vhl^∆/∆^Trp53^∆/∆^Rb1^∆/∆^ mice samples from *Frew* et al. showing the normalized reads of *Ranbp3l* [56]*.* ***, *p* < 0.001, Student’s *t* test. (E-F) RNA-Seq analysis from *Pili* et al. (E) [61] (**, *p <* 0.01, Student’s *t* test) and *Lai* et al. (F) [62] of normal tissue and collecting duct carcinoma (CDC) samples showing the read counts of *RANBP3L.*

**Additional file 10: Figure S5.** (A) Mean log_2_ expression of *NFAT5* in all TCGA RCC samples compared to normal tissue. Values were analyzed with a student’s-T test and are presented in a whiskers 1–99 percentile plot, ***, *p*<0.001. *n =* the number of available samples in the indicating TCGA cohort. (B) Among RCC subtypes *NFAT5* expression is downregulated in KIRC and KIRP. Values were analyzed with a student’s-T test and are represented in a whiskers 1–99 percentile plot, n.s., *p >* 0.05, **, *p <* 0.01, ***, *p<*0.001. *n =* the number of available samples obtained from TCGA.

**Additional file 11: Figure S6.** (A) RT-PCR of different RCC cell lines *for RANBP3L* and *GAPDH.* The cell lines were incubated in isoosmolar or hyperosmolar medium before RNA isolation. As a control normal kidney tissue and *GAPDH* was used. (B) Immunofluorescence images of different RCC cell lines showing NFAT5 nuclear translocation under hyperosmolar cultivation. Scale bar: 100 μm.

**Additional file 12: Figure S7.** (A, C, E) Plot of the wound density over time of 786–0 (A), Caki-1 (C) and Caki-2 (E). Migration assay was performed for 24–48 h. Values represent mean ± SEM (error bars). *n =* 2–3 (with 2–4 technical replicates). The relative migration change after 8 h (for 786–0) and after 16 h (for Caki-1 and Caki-2) was calculated by linear regression analysis using GraphPad 9 Prism. ***, *p <* 0.001, Student’s *t* test. (B, D, F). Proliferation analyzes of 786–0 (B), Caki-1 (D), and Caki-2 (F). Cells were cultivated in 96-well plates and the proliferation was measured by live-cell imaging using the IncuCyte S3 system taking an image every 4 h for 24–48 h. The relative cell density was calculated by IncuCyte S3 software and normalized to the cell density at 0 h. The data points were fitted by nonlinear exponential growth equation using GraphPad Prism to calculate the mean doubling times. Values represent mean ± SEM (error bars). *n =* 2–3 (with 2 technical replicates), ***, *p <* 0.001, Student’s *t* test.

## Data Availability

The gene expression data will be submitted to Gene Expression Omnibus.

## Abbreviations

4-OH-TM: 4-Hydroxytamoxifen
C57BL/6: C57 black 6
Cas9: RISPR-associated protein 9
CDC: Collecting duct carcinoma
CRISPR: Clustered regularly interspaced short palindromic repeats
DAPI: 4′,6-diamidino-2-phenylindole
DEG: Differential expressed genes
EMT: Epithelial-to-mesenchymal transition
FBS: Fetal bovine serum
Flx: Floxed
GEPIA2: Gene expression profiling interactive analysis
gRNA: Guide RNA
GSEA: Gene Set Enrichment Analysis
IGV: Integrative Genomics Viewer
KEGG: Kyoto Encyclopedia of Genes and Genomes
KICH: Kidney Renal chromophobe Cell Carcinoma
KIRC: Kidney Renal Clear Cell Carcinoma
KIRP: Kidney renal papillary cell carcinoma
PBS: Phosphate buffer saline
PBST: PBST plus 0,01% Triton
RCC: Renal cell carcinoma
RIPA: Radioimmunoprecipitation assay buffer
RT-qPCR: Reverse transcriptase- quantitative PCR
SV: SV40 early enhancer
TCGA: The Cancer Genome Atlas
TIDE: Tracking of Indels by Decomposition
