# Supplementary material for: Loss of RANBP3L leads to transformation of renal epithelial cells towards a renal clear cell carcinoma like phenotype
Source: J Exp Clin Cancer Res. 2021 Jul 7;40:226. doi: 10.1186/s13046-021-01982-y (PMC8265145; doi:10.1186/s13046-021-01982-y)
Supplement: Supplementary file 6 — Additional file 6: Figure S1. (A) Schematic representation of domain organization in RANBP3L and RANBP3 (AS sequence in Table S1). (B) Median gene expression of RANBP3 and RANBP3L in a bodymap from the gene expression profiling interactive analysis tool (GEPIA2) [33]. (C) Schematic representation of IMCD isolation and culturing from murine kidneys for next-generation sequencing (NGS). (D-E) Analysis of NFAT5 intensity in mpKCCD immunofluorescence images (D) and in IMCD cell immunofluorescence images (E) shown in Fig. 2A. Scale bar 100 μm. (F-G) The NFAT5 fluorescence intensities of cells were plotted on a line graph. Lines correspond to the relative fluorescence of cells marked with a white arrow. F corresponds to signal intensity in mpkCCD cell while G corresponds to signal intensity of IMCD cells. (H) TIDE analysis of N1 and N3 single clones reveal INDELs in the Nfat5 target sites [26]. (I) Selected distribution of RNA-Seq reads across the Nfat5 gene for control and 4-OH-TM treated cells shows loss of exon 4. (J) Screen capture of human RANBP3L promotor region from the ECR Browser website with evolutionary conserved regions (ECR) in the genomes of rat, mouse, canis, macaca and pan. The lines in pink (arrows ahead) show ECR between human and the indicated mammals [28]. (K) 800 bp region uptream from RANBP3L start site is conserved in the indicated mammals and contains one conserved NFAT5 consensus sequence (NNTTTCCA is indicated in yellow) and one non-conserved (nucleotides are indicated in red). (L) Relative promotor activity of all Ranbp3l promotor constructs normalized to the isoosmolar control (n = 4, each with duplicates). n.s. > 0.05, *, p < 0.05, **, p < 0.01. Student’s t test. [file 13046_2021_1982_MOESM6_ESM.docx]

**Figure S1:**

(A) Schematic representation of domain organization in RANBP3L and RANBP3 (AS sequence in table S1). (B) Median gene expression of RANBP3 and RANBP3L in a bodymap from the gene expression profiling interactive analysis tool (GEPIA2) [33]. (C) Schematic representation of IMCD isolation and culturing from murine kidneys for next-generation sequencing (NGS). (D-E) Analysis of NFAT5 intensity in mpKCCD immunofluorescence images (D) and in IMCD cell immunofluorescence images (E) shown in Fig. 2A. Scale bar 100µm. (F-G) The NFAT5 fluorescence intensities of cells were plotted on a line graph. Lines correspond to the relative fluorescence of cells marked with a white arrow. F corresponds to signal intensity in mpkCCD cell while G corresponds to signal intensity of IMCD cells. (H) TIDE analysis of N1 and N3 single clones reveal INDELs in the *Nfat5* target sites [25]. (I) Selected distribution of RNA-Seq reads across the *Nfat5* gene for control and 4-OH-TM treated cells shows loss of exon 4. (J) Screen capture of human *RANBP3L* promotor region from the ECR Browser website with evolutionary conserved regions (ECR) in the genomes of rat, mouse, canis, macaca and pan. The lines in pink (arrows ahead) show ECR between human and the indicated mammals [27]. (K) 800 bp region uptream from *RANBP3L* start site is conserved in the indicated mammals and contains one conserved NFAT5 consensus sequence (NNTTTCCA is indicated in yellow) and one non-conserved (nucleotides are indicated in red). (L) Relative promotor activity of all *Ranbp3l* promotor constructs normalized to the isoosmolar control (n=4, each with duplicates). n.s. > 0.05, *, p < 0.05, **, p < 0.01. Student’s t test.
