# Supplementary material for: Loss of RANBP3L leads to transformation of renal epithelial cells towards a renal clear cell carcinoma like phenotype
Source: J Exp Clin Cancer Res. 2021 Jul 7;40:226. doi: 10.1186/s13046-021-01982-y (PMC8265145; doi:10.1186/s13046-021-01982-y)
Supplement: Supplementary file 7 — Additional file 7: Figure S2. (A) Phase contrast images of Scr, R1 and R3 mpkCCD cells with 300 mosmol/kg and 600 mosmol/kg. Arrowheads indicate colony-detaching cells. Scale bar: 400 μm (B) Fluorescence of F-Actin staining in Scr, R1 and R3 mpkCCD cells using Phalloidin coupled to Alexa-568 and DAPI for nucleus visualization (Scale bar: 100 μm). (C-D) TIDE (Tracking of Indels by Decomposition) analysis of R1 (C) and R3 (D) single clone revealing INDELs in Ranbp3l target site [26]. (E-F) Proliferation analyses of Scr, R1 and R3 mpkCCD cells. Cells were cultivated in 96-well plates and the proliferation was measured by live-cell imaging using the IncuCyte S3 system taking an image every 4 h for 24 h. The relative cell density was calculated by IncuCyte S3 software and normalized to the cell density at 0 h. The data points were fitted by nonlinear exponential growth equation using GraphPad Prism to calculate the mean doubling times. Values represent mean ± SEM (error bars). n = 3 (with 4 technical replicates) n.s.,p > 0.05, **, p < 0.01; ***, p < 0.001, 1-way ANOVA. [file 13046_2021_1982_MOESM7_ESM.docx]

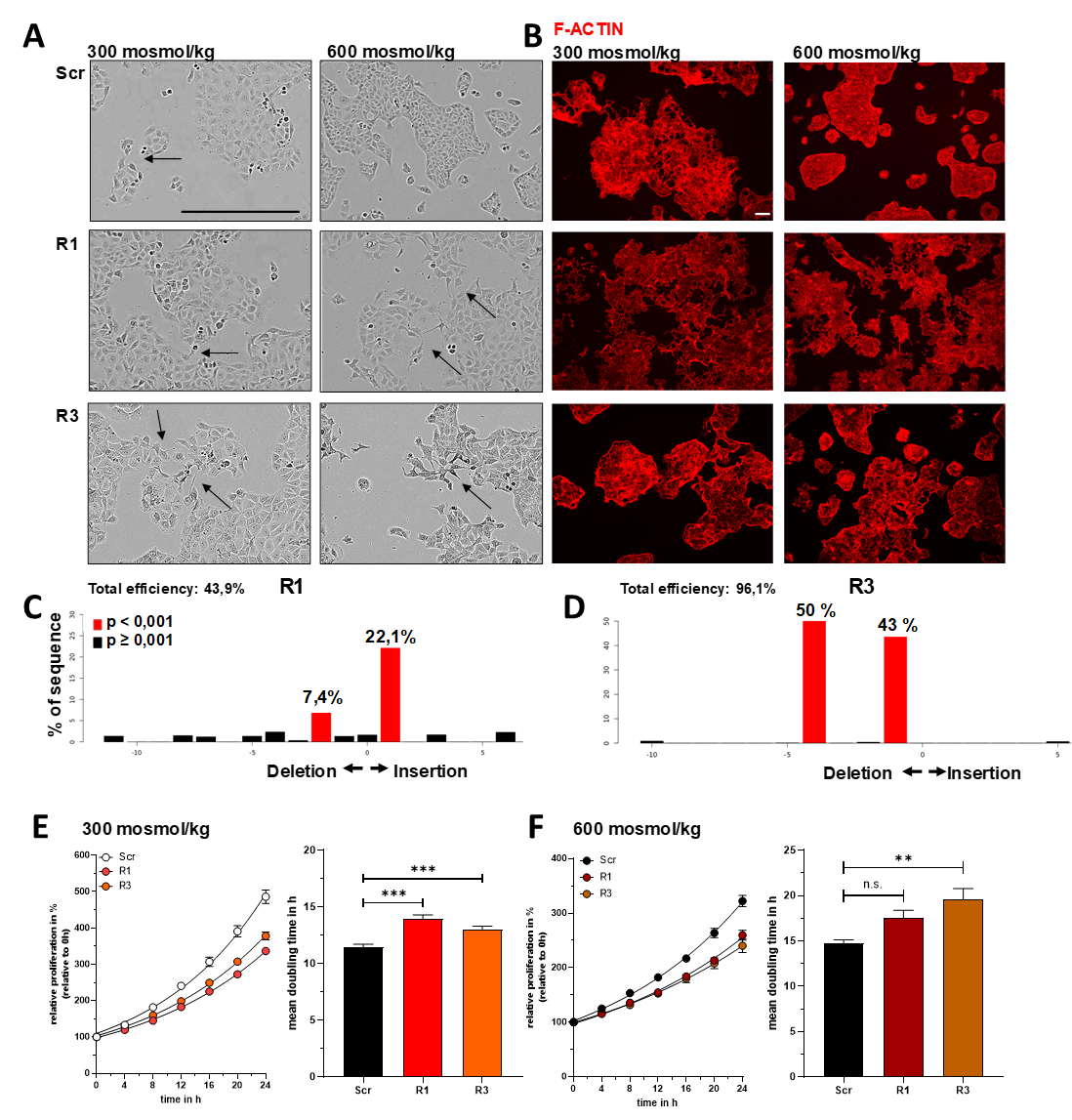


**Figure S2.**

(A) Phase contrast images of Scr, R1 and R3 mpkCCD cells with 300 mosmol/kg and 600 mosmol/kg. Arrowheads indicate colony-detaching cells. Scale bar: 400µm (B) Fluorescence of F-Actin staining in Scr, R1 and R3 mpkCCD cells using Phalloidin coupled to Alexa-568 and DAPI for nucleus visualization (Scale bar: 100 µm). (C-D) TIDE (Tracking of Indels by Decomposition) analysis of R1 (C) and R3 (D) single clone revealing INDELs in *Ranbp3l* target site [25]. (E-F) Proliferation analyses of Scr, R1 and R3 mpkCCD cells. Cells were cultivated in 96-well plates and the proliferation was measured by live-cell imaging using the IncuCyte S3 system taking an image every 4 h for 24 h. The relative cell density was calculated by IncuCyte S3 software and normalized to the cell density at 0 h. The data points were fitted by nonlinear exponential growth equation using GraphPad Prism to calculate the mean doubling times. Values represent mean ± SEM (error bars). n = 3 (with 4 technical replicates) n.s.,p > 0.05, **, p ˂ 0.01; ***, p ˂ 0.001, 1-way ANOVA.
