# Supplementary material for: Loss of RANBP3L leads to transformation of renal epithelial cells towards a renal clear cell carcinoma like phenotype
Source: J Exp Clin Cancer Res. 2021 Jul 7;40:226. doi: 10.1186/s13046-021-01982-y (PMC8265145; doi:10.1186/s13046-021-01982-y)
Supplement: Supplementary file 8 — Additional file 8: Figure S3. (A-B) GSEA of RANBP3L dependent genes shows enrichment of the REACTOME Paythways “degradation of extracellular matrix” and “collagen formation” in RANBP3L-KO cells. (C) Mean log2 fold change of 1771 overlapping genes with prognostic favorable (blue), unfavorable (yellow) and uncategorized (black) genes in RCC. (D-E) RT-qPCR validation of different RANBP3L-KO clones (each in triplicates) for (D) Sparc, Col4a1, Col4a2 and (E) Sh3gl2, Aldh2 and Hoxa7. Values represent mean ± SEM (error bars), n.s. > 0.05, *, p < 0.05, **, p < 0.01, Student’s t test. (F-G) Pearson correlation analyses of RANBP3L expression and the upregulated (F, not including ADGRA2) and downregulated (G) signature expression in KIRC using the TCGA data set. [file 13046_2021_1982_MOESM8_ESM.docx]

**Figure S3:**

(A-B) GSEA of RANBP3L dependent genes shows enrichment of the REACTOME Paythways “degradation of extracellular matrix” and “collagen formation” in RANBP3L-KO cells. (C) Mean log_2_ fold change of 1771 overlapping genes with prognostic favorable (blue), unfavorable (yellow) and uncategorized (black) genes in RCC. (D-E) RT-qPCR validation of different RANBP3L-KO clones (each in triplicates) for (D) *Sparc*, *Col4a1*, *Col4a2* and (E) *Sh3gl2*, *Aldh2* and *Hoxa7*. Values represent mean ± SEM (error bars), n.s. > 0.05, *, p < 0.05, **, p < 0.01, Student’s t test. (F-G) Pearson correlation analyses of *RANBP3L* expression and the upregulated (F, not including *ADGRA2*) and downregulated (G) signature expression in KIRC using the TCGA data set.
