# Supplementary material for: Loss of RANBP3L leads to transformation of renal epithelial cells towards a renal clear cell carcinoma like phenotype
Source: J Exp Clin Cancer Res. 2021 Jul 7;40:226. doi: 10.1186/s13046-021-01982-y (PMC8265145; doi:10.1186/s13046-021-01982-y)
Supplement: Supplementary file 9 — Additional file 9: Figure S4. (A-C) Somatic mutation analysis of RANBP3L in KIRC (A), KICH (B) and KIRP (C). (D) RNA-Seq analysis of WT (wildtype) and Vhl∆/∆Trp53∆/∆Rb1∆/∆ mice samples from Frew et al. showing the normalized reads of Ranbp3l [56]. ***, p < 0.001, Student’s t test. (E-F) RNA-Seq analysis from Pili et al. (E) [61] (**, p < 0.01, Student’s t test) and Lai et al. (F) [62] of normal tissue and collecting duct carcinoma (CDC) samples showing the read counts of RANBP3L. [file 13046_2021_1982_MOESM9_ESM.docx]

**Figure S4:**

(A-C) Somatic mutation analysis of *RANBP3L* in KIRC (A), KICH (B) and KIRP (C). (D) RNA-Seq analysis of WT (wildtype) and Vhl^∆/∆^Trp53^∆/∆^Rb1^∆/∆^ mice samples from *Frew et al.* showing the normalized reads of *Ranbp3l* [63]*.* ***, p < 0.001, Student’s t test. (E-F) RNA-Seq analysis from *Pili et al.* (E) [59] (**, p < 0.01, Student’s t test) and *Lai et al.* (F) [61] of normal tissue and collecting duct carcinoma (CDC) samples showing the read counts of *RANBP3L.*
