# Supplementary material for: Loss of RANBP3L leads to transformation of renal epithelial cells towards a renal clear cell carcinoma like phenotype
Source: J Exp Clin Cancer Res. 2021 Jul 7;40:226. doi: 10.1186/s13046-021-01982-y (PMC8265145; doi:10.1186/s13046-021-01982-y)
Supplement: Supplementary file 10 — Additional file 10: Figure S5. (A) Mean log2 expression of NFAT5 in all TCGA RCC samples compared to normal tissue. Values were analyzed with a student’s-T test and are presented in a whiskers 1–99 percentile plot, ***, p<0.001. n = the number of available samples in the indicating TCGA cohort. (B) Among RCC subtypes NFAT5 expression is downregulated in KIRC and KIRP. Values were analyzed with a student’s-T test and are represented in a whiskers 1–99 percentile plot, n.s., p > 0.05, **, p < 0.01, ***, p<0.001. n = the number of available samples obtained from TCGA. [file 13046_2021_1982_MOESM10_ESM.docx]

**Figure S5:**

(A) Mean log_2_ expression of *NFAT5* in all TCGA RCC samples compared to normal tissue. Values were analyzed with a student’s-T test and are presented in a whiskers 1-99 percentile plot, ***, p˂0.001. n = the number of available samples in the indicating TCGA cohort. (B) Among RCC subtypes *NFAT5* expression is downregulated in KIRC and KIRP. Values were analyzed with a student’s-T test and are represented in a whiskers 1-99 percentile plot, n.s., p> 0.05, **, p< 0.01, ***, p˂0.001. n = the number of available samples obtained from TCGA.
