# Supplementary material for: Loss of RANBP3L leads to transformation of renal epithelial cells towards a renal clear cell carcinoma like phenotype
Source: J Exp Clin Cancer Res. 2021 Jul 7;40:226. doi: 10.1186/s13046-021-01982-y (PMC8265145; doi:10.1186/s13046-021-01982-y)
Supplement: Supplementary file 11 — Additional file 11: Figure S6. (A) RT-PCR of different RCC cell lines for RANBP3L and GAPDH. The cell lines were incubated in isoosmolar or hyperosmolar medium before RNA isolation. As a control normal kidney tissue and GAPDH was used. (B) Immunofluorescence images of different RCC cell lines showing NFAT5 nuclear translocation under hyperosmolar cultivation. Scale bar: 100 μm. [file 13046_2021_1982_MOESM11_ESM.docx]

**Figure S6:**

(A) RT-PCR of different RCC cell lines *for RANBP3L* and *GAPDH.* The cell lines were incubated in isoosmolar or hyperosmolar medium before RNA isolation. As a control normal kidney tissue and *GAPDH* was used. (B) Immunofluorescence images of different RCC cell lines showing NFAT5 nuclear translocation under hyperosmolar cultivation. Scale bar: 100µm.
