# Supplementary material for: Loss of RANBP3L leads to transformation of renal epithelial cells towards a renal clear cell carcinoma like phenotype
Source: J Exp Clin Cancer Res. 2021 Jul 7;40:226. doi: 10.1186/s13046-021-01982-y (PMC8265145; doi:10.1186/s13046-021-01982-y)
Supplement: Supplementary file 12 — Additional file 12: Figure S7. (A, C, E) Plot of the wound density over time of 786–0 (A), Caki-1 (C) and Caki-2 (E). Migration assay was performed for 24–48 h. Values represent mean ± SEM (error bars). n = 2–3 (with 2–4 technical replicates). The relative migration change after 8 h (for 786–0) and after 16 h (for Caki-1 and Caki-2) was calculated by linear regression analysis using GraphPad 9 Prism. ***, p < 0.001, Student’s t test. (B, D, F). Proliferation analyzes of 786–0 (B), Caki-1 (D), and Caki-2 (F). Cells were cultivated in 96-well plates and the proliferation was measured by live-cell imaging using the IncuCyte S3 system taking an image every 4 h for 24–48 h. The relative cell density was calculated by IncuCyte S3 software and normalized to the cell density at 0 h. The data points were fitted by nonlinear exponential growth equation using GraphPad Prism to calculate the mean doubling times. Values represent mean ± SEM (error bars). n = 2–3 (with 2 technical replicates), ***, p < 0.001, Student’s t test. [file 13046_2021_1982_MOESM12_ESM.docx]

**Figure S7:**

(A, C, E) Plot of the wound density over time of 786-0 (A), Caki-1 (C) and Caki-2 (E). Migration assay was performed for 24-48 h. Values represent mean ± SEM (error bars). n = 2-3 (with 2-4 technical replicates). The relative migration change after 8 h (for 786-0) and after 16 h (for Caki-1 and Caki-2) was calculated by linear regression analysis using GraphPad 9 Prism. ***, p ˂ 0.001, Student’s t test. (B, D, F). Proliferation analyzes of 786-0 (B), Caki-1 (D), and Caki-2 (F). Cells were cultivated in 96-well plates and the proliferation was measured by live-cell imaging using the IncuCyte S3 system taking an image every 4 h for 24-48 h. The relative cell density was calculated by IncuCyte S3 software and normalized to the cell density at 0 h. The data points were fitted by nonlinear exponential growth equation using GraphPad Prism to calculate the mean doubling times. Values represent mean ± SEM (error bars). n = 2-3 (with 2 technical replicates), ***, p ˂ 0.001, Student’s t test.
